# What leads to innovation in mental healthcare? Reflections on clinical expertise in a bureaucratic age

**DOI:** 10.1192/bjb.2018.14

**Published:** 2018-10

**Authors:** Neil Armstrong

**Affiliations:** University of Oxford, UK

## Abstract

This paper considers a witness seminar in which healthcare professionals discussed working on an acute admissions ward run along therapeutic community lines from the 1960s to the 1980s. Participants remarked that older styles of working are ‘unimaginable’ today. This paper discusses why. Literature from the humanities and social sciences suggest healthcare is reactive, reflecting wider cultural changes, including a preference for a more bureaucratic, standardised, explicit style of reasoning and a high valuation of personal autonomy. Such a reflection prompts questions about the nature of professional expertise, the role of evidence and the importance of the humanities and social sciences.

**Declaration of interest:**

None.

## Professional autonomy as a source of authority

It is tempting to think of clinical expertise as autonomous. Indeed, it appears to be an essential part of the role of mental healthcare clinicians that they do not merely reflect or reproduce everyday ideas and assumptions about mental disorders and their treatment, but bring something distinctive to the table. This distinctive contribution is the result of intensive training, painstaking clinical experience and robust scientific research. Understood in this way, collective professional sovereignty seems to be a critical ingredient in differentiating experts from the lay public. It is on the basis of this differentiation that expert opinion may be seen as deserving more weight than the views of a non-specialist. This might suggest that a contrast between expert knowledge and lay knowledge is both necessary and desirable. Educating patients, their family and friends, and the wider public typically means challenging lay assumptions and replacing them with clinically authorised knowledge. This has been the way that campaigns against stigma have been conceived.^1^ Similarly, ‘psychoeducation’, which is deemed to be a possibly beneficial adjunct to care for people receiving treatment for mental disorders, is designed by clinicians and intended for the patients, and not the other way round.

There a number of reasons that disciplinary autonomy might be a useful and attractive notion. Autonomy contributes to authority in a setting where authority is both justified and necessary. However, these considerations might encourage a lack of attention to the real status of mental healthcare expertise. This autonomy may not be quite as it appears. I do not want to suggest here that the collective professional sovereignty of mental healthcare clinicians is entirely false, illusory or illegitimate. Instead, I want to make a more cautious argument that, in practice, professional expertise is deeply reactive. Rather than being the sole active agent, mental healthcare professionals as a group are acted upon by wider social and cultural forces. Factors external to professional bodies can shape expertise and practice in ways that are critical and yet difficult to detect.

The views of the general public about, say, what might count as a pathological personal trait and how this might be differentiated from what might better be thought of as eccentric, shape clinical judgement. In practice, any other relationship would be rather odd: mental healthcare professionals are in a position to develop common-sense thinking about distress and madness, but if it were to diverge too far from public opinion, it would be likely to lose its claim to authority. A concrete example might be homosexuality. In the 1960s and 1970s, this was first modified and then dropped as a diagnostic category, not because of research findings, but because of wider shifts in public opinion.^2^^,^^3^ This is instructive. However much it is predicated on science, the authority of mental healthcare practitioners also rests on popular consent. A widespread loss of confidence in mental healthcare would mean an existential challenge for the discipline as a whole. This complex and two-way relationship between expert knowledge and common sense is not unique to mental healthcare. In a major study of scientific and folk thinking about biology, Atran found continuity between ‘ordinary thinking’ categories describing vegetation and those of the biological sciences, such that each can learn from the other.^4^

## Healthcare and its cultural surround

An important point for this discussion is that the external factors that shape clinical work are not always as simple as popular views about sexuality, or the acceptability of unusual character traits. More subtle influences are at work. Basic clinical categories and practices that clinicians might typically think of as arising out of autonomous clinical expertise seem to resonate with widely discussed social and cultural processes. These processes are moral as much as scientific, dealing with values, commitments and orientations. We might wonder whether the cart is pulling the horse or, indeed, which is which.

Take, for example, the notions of risk, accountability, responsibility and anxiety. All figure large in clinical practice. Drawing on the work of Beck, Giddens argues that accountability is linked to risk and anxiety, forming part of what he calls the ‘contours of high modernity’ in which personhood is being ‘reshaped’.^5^^,^^6^ It appears that this high modernity shapes medicine as much as medicine shapes high modernity. Giddens notes how individuals increasingly see life as containing risks, and that dealing with risk and controlling the future is one of the ‘core aspects of modernity’. Critically, according to him, dealing with risk is now an individual challenge. The modern individual seeks to personally equip himself or herself by means of gaining knowledge to enable autonomous decision-making. These ideas seem to be reflected in the way that patient-centred care or patient empowerment is conceptualised. We might conclude that the personalisation of care and the goal of patient empowerment are part of modernity rather than arising out of autonomous clinical reason.

In a similar way, for many commentators, what knowledge is and what might count as good reasons for a decision are changing. For example, it is increasingly the case that a good decision is one that might be defended in an impersonal way. In *Trust in Numbers*, Porter makes a distinction between what he calls ‘disciplinary objectivity’ and ‘mechanical objectivity’.^7^ The former arises out of a consensus of experts. As it consists of expert judgement, it demands trust. By contrast, mechanical objectivity is derived from the use of quantified data and the following of rules. This appears impartial, rigorous and unbiased.

Porter suggests that much of the appeal of quantitative, mechanical objectivity is ‘mythical’ and confused. It is nonetheless very attractive. Part of the reason for this is that it appears impersonal, in that data and algorithms replace personal judgement. According to Porter, underlying mechanical objectivity is an ethic that ‘rules should rule’, rather than people ruling. He puts it like this: ‘Scientific objectivity thus provides an answer to a moral demand for impartiality and fairness. Quantification is a way of making decisions without seeming to decide’^7^ (p. 8). These epistemic preferences shape our sense of what expertise consists of. Expertise is taken to be a capacity for explicit and auditable decision-making. Good decisions are impartial and fair, which is signalled by the erasure of the decision maker.

These trends are widely recognised. A great deal of professional work today is being reconfigured. Whether it is education, banking, the military or international development, expertise is becoming formalised and ‘managerialised’, reinterpreted as a sequence of explicit decisions, capable of being scrutinised externally, and underwritten by the mechanical objectivity of evidence and policy. Graeber talks of the ‘era of total bureaucratization’ in which standardised forms of knowledge are reductive and yet dominant, unsatisfactory and yet overpowering.^8^ The effects of this are ubiquitous, but particularly found in medicine. Porter^7^ comments that:

In public even more than in private affairs, expertise has more and more become inseparable from objectivity. Indeed, to recur to the previous example, it is in part because the relation of physician to patient is no longer a private one – due to the threat that it might be opened up in a courtroom – that instruments have become central to almost every aspect of medical practice (p. 7).

Harrison argues that the rise of evidence-based medicine and the introduction of market mechanisms in the National Health Service has led to a change in the way that clinical knowledge and expertise are conceived of, a transition away from instinct or personal judgement, and towards propositional knowledge and instrumental rationality.^9^

## Clinical memory as a source of knowledge

One way of thinking through these issues is to attend to the experiences of staff whose careers span different working styles. The Phoenix Unit was an acute admissions ward run according to the therapeutic community concept at the Littlemore Hospital in Oxford. It was set up by Bertram Mandelbrote in 1959 and closed in 1996. The ethos of the Phoenix was to explore the behaviours and feelings of residents through community life and, in particular, in group settings. Daily community groups formed the centre of care and were supplemented by working groups, occupational therapy, crisis groups and relatives’ groups. Over time, the ward became well known and attracted considerable interest.

A reunion of Phoenix Unit staff members took place on 19 October 2016 in the Barns Conference Centre, part of the Planned Environment Therapy Trust (PETT), near Cheltenham in Gloucestershire. There were 23 participants, including psychiatrists, nurses, a psychologist, a social worker, an occupational therapist and an art therapist. The reunion consisted of six recorded sessions. After a 30-min introductory session, the group split in two, with each subgroup talking for about 80 min. There followed three sessions involving all participants, totalling almost 2.5 h. So, the total record is a little short of 6 h discussion. All the recordings were transcribed, and both the recordings and the transcriptions are archived by the PETT. There was a lot of mutual recognition among the participants. Many were happy to be reunited with former colleagues. Some participants were longstanding friends, others less known. Participants were mostly confident about speaking in a large group. In fact, participating in group discussion might be understood as a facet of clinical skill. No formal methodology was adopted to analyse the material. As might be appropriate, given the themes of the paper, the author relies on his own judgement about the discussions, rather than seeking authority through algorithm or quantification.

Staff remember the Phoenix as ‘very 60s’ and ‘very permissive’. A culture of openness extended to patients, ex-patients, families and staff. ‘We were all part of it’, said one. The atmosphere on the ward was described as ‘apparent chaos’ or ‘chaos’, and by one person as ‘the maddest place I've ever seen’. Hierarchies were flattened, professional roles blurred, and staff understood themselves to work ‘alongside’ patients as equals. Interaction was relaxed and informal. Patients were encouraged to express themselves. One participant said people ‘came to be mad’. Another remarked that staff ‘allowed behaviour’. Expertise was not located solely with the professionals. Patients helped other patients, and staff learned from patients. It was said that ‘Everyone was a therapist, including the patients’.

The unit gave staff huge amounts of freedom. Discretion and professional judgement were favoured over routines and standardised rationales. Care seemed to rest on spontaneous creative decisions. A spate of broken windows led to patients being paid to break windows. As one participant put it: ‘there was no plan … no structured response’ to events. There was a stress on ‘carrying the culture’ but less emphasis on note-taking. Written documentation was minimal. It is telling that key therapeutic events took a form that, to the bureaucratised imagination, sounds like an oxymoron: ‘agendaless’ meetings.

Diagnosis and targeted medications seem to have played a small part. The flexible, non-medicalised culture of the unit was described as helping staff to understand patients as people, rather than carriers of impersonal disorders. It helped one participant to see ‘the person behind the patient’, and another to see that ‘we're all human beings’. In addition, a less medical approach to distress reduced the superordination of doctors and meant all the staff members had a part to play. The opinions of cleaners, for example, were frequently sought. However, patients had to commit to the process. Group attendance was compulsory, and patients who didn't engage might be considered for discharge. And when drugs were prescribed, even if it was to ‘contain’ excessive mania or anxiety, rather than to treat a disorder, they were in what one psychiatrist dubbed the ‘monster doses’ typical of the period.

## Understanding the Phoenix from the perspective of the present

The witness seminars are remarkable because they show how profoundly and how rapidly care has changed. My suggestion is that this change reflects wider cultural processes. It isn't that accurate forms of measurement showed that the unit was less effective, or less safe or more expensive than contemporary arrangements (although it may have been some or all of those things). Rather, the cultural surround changed such that less patterned practice seems less professional. In Porter's terms, improvised, intuitive exchanges between staff and patients are not constitutive of mechanical authority.

Several participants remarked that problems with the production of paperwork are why such a unit could not be imagined today. People on the Phoenix ‘were bad at taking notes’, which made it ‘the opposite of what is going on now’. It is hard to see how the kinds of activities described by the participants could be recorded in an economical and standardised way. Unpatterned, improvised and complex activity is difficult to document. In highly bureaucratised times, care that can't be documented is not professional. Personal judgement and the ‘culture’ of the institution are weak justifications for treatment.

Moreover, care was more organised around personal development than patient empowerment. As a permissive setting in which individual preference trumped rules, the unit might be seen as the vanguard of individualism. Yet care in the Phoenix was modelled around goals that reflected a more social and less individualistic conception of personhood and human flourishing. The reunion suggests something further: that seeing people as social beings might be difficult to square with bureaucratic culture. This social dimension of mental health might fall somewhere beyond what we might expect an accountable bureaucracy to successfully address. To use Giddens's phrase, the Phoenix shared few of the contours of high modernity.

The influence of all of this on staff members appears mixed. Routinisation can protect staff members from the pressures of deliberation and personal responsibility. Rule-following requires less of a staff member than thinking problems through afresh. In Porter's terms, impersonal ‘mechanical objectivity’ erases the individual clinician. By contrast, spontaneity is hard work. As a result, staff seem to have found it extremely taxing to work at the Phoenix, but also enormously educative and influential. More was expected of them, and more was gained by them. Staff could flourish, but they could also burn out. Working at the Phoenix led to self-exploration that was at once therapeutic and intensely stressful. The impression created by the seminar participants is that standardisation and mechanical objectivity protect clinicians. It appears that this comes at a cost: clinicians who are shielded by rules have reduced opportunities for learning.

## Conclusion

The recollections of the Phoenix staff resemble in striking ways widespread changes in professional culture discussed in the humanities and social science literature. A trend towards more bureaucratic working practices, and for expertise to consist of a capacity to adopt a standardised, explicit style of reasoning, are widely observed in the literature and were widely remarked upon by the Phoenix staff. This suggests that innovations in clinical practice are, to a degree at least, determined by cultural factors external to the mental healthcare professions. This is not to claim that the only source of innovation is the cultural surround. However, clinical practice, styles of service delivery and planning, and even notions of professional expertise and patient health are being reconfigured in ways that appear to be part of a wider transformation of professional lives. Whether we like it or not, we live in bureaucratic times. Bureaucratic values increasingly enter professional domains, blurring distinctions between formerly separate bodies of expertise. Clinical sensibilities about risk, for example, resemble those of a policy maker or bureaucrat.^10^ It is telling that good mental health is increasingly framed in terms of personal autonomy and social and economic independence. There is nothing specifically clinical about these categories. Instead, they are rather open and flexible notions, which may be transferred across professional frontiers. They seem to naturally belong to Giddens's high modernity.

This might seem an unappealing state of affairs. As I tried to suggest in the first part of the paper, one reason for unease might be that admissions of cultural influence can be seen to undermine clinical authority. I suggest that this need not be the case. Further, I argue that such influence is anyway inevitable. The relatively unstructured and hard-to-document working practices in the Phoenix unit would be unthinkable today not for reasons of evidence of effectiveness, safety or value for money. Instead, they look unprofessional. They would be difficult to audit. We might imagine they would stick out in documentation presented to the Care Quality Commission, or to the local clinical commissioning group.

This has a number of implications, not least regarding how to understand innovation in medical practice. If a cultural impetus as identified by Porter, for ‘rules to rule’ and not people to rule, leads to shifts in clinical practice such as those described by the Phoenix staff members, what are we to make of other accounts of innovation, such as those that refer to accumulating evidence? If the old style of working is just out of epistemic fashion, where does that leave the rise of evidence-based medicine? Perhaps Grimley Evans is right when he comments, acerbically, that part of the appeal of evidence-based medicine is that it offers ‘total managerial control’ of healthcare.^11^

These reflections also prompt questions regarding the status of ‘expertise by experience’. We might suspect that the kind of expertise that patients have by virtue of first-person experiences of health and healthcare more closely resembles the expertise of the Phoenix Ward staff than that of contemporary clinicians. Do current epistemic sensibilities prejudice us against expertise by experience? Do we undervalue expertise by experience because of quite recent changes in how we understand the nature of expertise itself?

These questions are of significant clinical weight. Yet they are more the province of the humanities and social science disciplines, not least anthropology and philosophy, than the disciplines that make up mainstream mental healthcare research. Mental healthcare is increasingly the subject of investigation by disciplines whose starting assumptions and methods are not those of medicine. This appears to be an opportunity, not a threat. If the reunion attendees tell us anything, it is of the potential value of the contribution of the humanities and social sciences to the investigation of biomedicine.

## About the author

**Neil Armstrong** is a Lecturer in Anthropology at Magdalen College, University of Oxford, UK.
